# A population-based screening for hepatitis C antibodies and active infection using a point-of-care test in a low prevalence area

**DOI:** 10.1371/journal.pone.0228351

**Published:** 2020-02-11

**Authors:** Ângela Carvalho-Gomes, Almudena Cubells, Carmina Pallarés, Vanessa Hontangas, Isabel Conde, Tomasso Di Maira, Salvador Peiró, Gabriel Sanfélix-Gimeno, F. Xavier López-Labrador, Marina Berenguer

**Affiliations:** 1 Liver Transplantation and Hepatology Laboratory, Instituto de Investigación Sanitaria La Fe, València, Spain; 2 CIBERehd, Centro de Investigación Biomédica en Red en Enfermedades Hepáticas y Digestivas, Instituto de Salud Carlos III, Madrid, Spain; 3 Liver Transplantation and Hepatology Unit, Hospital Universitario y Politécnico La Fe, València, Spain; 4 Health Services Research Area, Fundación para el Fomento de la Investigación Sanitaria y Biomédica de la Comunidad Valenciana (FISABIO Public Health), València, Spain; 5 Virology Laboratory, Joint Units in Genomics and Health and Infection and Health, Fundación para el Fomento de la Investigación Sanitaria y Biomédica de la Comunidad Valenciana (FISABIO Public Health) / Universitat de València, València, Spain; 6 CIBEResp, Centro de Investigación Biomédica en Red en Epidemiología y Salud Pública, Instituto de Salud Carlos III, Madrid, Spain; 7 Department of Medicine, Universitat de València, València, Spain; Centers for Disease Control and Prevention, UNITED STATES

## Abstract

**Background:**

Data on the true prevalence of hepatitis C virus (HCV) infection in the general population is essential. We evaluated a program implementing free universal HCV screening using a non-invasive point-of-care test (POCT) (OraQuick-HCV rapid test) in oral fluid in an urban area in Valencia, South-Eastern Spain.

**Methods:**

A cross-sectional study was performed during 2015–2017. Free HCV screening was offered by regular mail to 11,500 individuals aged 18 and over, randomly selected from all census residents in the Health Department. All responding participants filled in a questionnaire about HCV infection risk factors and were tested in their tertiary Hospital. In those with a positive POCT, results were confirmed by enzyme-immunoassay and HCV-RNA.

**Results:**

1,206 persons agreed to participate (response rate: 11.16%). HCV antibodies were detected in 19 (1.60%) cases (age-sex standardized rate: 1.31%; 95%CI: 0.82–2.07), but only 8 showed positive HCV-RNA (age-sex standardized rate: 0.56%; 95%CI: 0.28–1.14). The majority (89%) of the cases were born before 1965 and 74% had at least one known risk factor for HCV infection. All anti-HCV positive individuals were already aware of their infection, and no undiagnosed cases were detected. The performance of the POCT was excellent for detecting active infection.

**Conclusions:**

These preliminary data suggest that HCV population screening with a POCT is feasible but, in our setting, mailing recruiting is not effective (11% response rate). The low prevalence of HCV antibodies and active infection in the participant population (with no new diagnoses made) suggests that, in our setting, underdiagnosis may be uncommon.

## Introduction

Chronic Hepatitis C Virus (HCV) infection represents a considerable economic burden on health-care systems.[1–3] A reliable estimation of the actual prevalence of HCV infection is needed to meet the World Health Organization first action plan.[4] In Spain, neither the current prevalence of HCV nor the proportion of undiagnosed cases are well-known.[5,6,7,8] Most studies on HCV prevalence in Spain have been conducted using the classical gold standard immunoassays (EIA/CIA) on serum or plasma samples and have focused on small portions of the population, often those considered to be at high risk [7,8]. The applicability of standard EIA/CIA assays for indiscriminate population screening is hampered by the requirement for venipuncture and specialist laboratory intervention. Screening with point-of-care tests (POCT) guarantees a rapid delivery of information and allows clinical decisions to be taken in a timely manner. One of them (OraQuick HCV) has been approved by the FDA and the EU for the diagnosis of HCV antibodies in venous blood, fingerstick, or oral mucosal transudate (OMT) [9,10]. This test is minimally (fingerstick) or non-invasive (OMT), simple to perform and interpret, and has shown a sensitivity and specificity equal or even superior to the gold standard (EIA/CIA) [11,13], making HCV screening in non-hospital settings feasible [8,10–14].

The OraQuick HCV rapid test has been used in several countries in small pilot studies to evaluate screening methods for the general population and/or specific risk groups [14–16]. We recently reported the performance of the test as a diagnostic technique for a rapid detection of anti-HCV antibodies in OMT and fingerstick blood samples. In our hands, the clinical sensitivity and specificity of the OraQuick HCV rapid test in OMT was up to 99.1% and 100%, respectively [13]. Thus, screening with point-of-care tests (POCT) represents an alternative to venous blood for population screening of active HCV infection in order to increase the number of tested subjects, and to include difficult-to-reach populations. [12–14,17]

We report a mailing recruiting intervention for universal HCV screening with a POCT, targeting the general population of a health department in the city of Valencia, south-eastern Spain, to estimate the prevalence of anti-HCV, active HCV infection, and undiagnosed cases at the population level.

## Material and methods

### Study design

We conducted a cross-sectional universal screening study in the general adult population of a Healthcare Department in Valencia (Spain), from September 2015 to October 2017 using the OraQuick anti-HCV rapid test followed, if positive, by gold-standard immunoassay (CIA) and characterized HCV positive cases including patient and virus characteristics.

### Setting

The Valencia La Fe Health Department is part of the Valencia Public Healthcare System, a healthcare network territorially organized providing universal healthcare coverage, free of charge except for a co-payment for drugs dispensed outside hospital, for about 95% of the residents in the Valencia Community. The Valencia Healthcare System has an information system with a unique identifier of all people covered (SIP, by its Spanish acronym), linking various administrative data including age, sex, address and the assigned hospital district. From this system, successive random samples of people assigned to our Hospital District were extracted by the informatics office of our Health department. In 2015, the districts assigned to “La Fe Department” included 153,318 inhabitants aged 18 and over, served by one public university hospital and several primary healthcare centres providing inpatient and outpatient care to all residents in its demarcation.

### Population and sample

The target population was composed of all individuals aged 18 and over covered by the Valencia Public Healthcare System in the Healthcare Department. Assuming an HCV prevalence of about 2%, we estimated a sample size for HCV screening of about 6,000 individuals (n = 5,934 with a 95% confidence interval and 0.5% precision), expecting a 50% response rate based in our experience with other surveys. Due to low recruitment rates, we later increased the number of individuals we attempted to contact in a second sample population of 30,000 target adults randomly selected by age and sex strata as the previous sample (see Results). From July 2015 to October 2017 a proposal for free screening was offered to 11,500 individuals in successive waves of ordinary mail. Target individuals were adults over 18 years of age, randomly selected by age and sex strata in order to obtain a participant sample close to the overall district population. An informative leaflet of the HCV screening procedure was included in the mailing.

### Inclusion and exclusion criteria

Inclusion criteria were persons of 18 years and over who had received a proposal by random population sampling and agreed to participate in the study by signing the informed consent form. Exclusion criteria were those who did not meet the inclusion criteria, and/or refused to participate.

### Risk factors questionnaire/demographic characteristics

Before performing the OraQuick HCV rapid test, all participants were invited to sign the informed consent form and fill out a questionnaire regarding demographic characteristics and risk factors for HCV infection, based on the World Health Organization *Global Hepatitis Report* [4] (Table 1). Information regarding viral hepatitis, HCV infection, risk factors associated with HCV transmission, and potential consequences of HCV infection were provided to all participants.

**Table 1:** Set of risk factors used in the questionnaire, as based on the World Health Organization Global Hepatitis Report [4].

**General risk factors**
Parenteral drug use (present or past)
HIV infection
Haemophilia
Haemodialysis
Blood transfusion or organ transplantation or coagulation factors administration before 1992
Elevation of liver enzymes above the upper normal limit
**Other HCV potential exposure**
HCV-infected relatives
Health care worker
Piercing
Tattooing
More than one lifetime partner

### OraQuick HCV rapid antibody test

The OraQuick HCV rapid test is a single-use lateral flow indirect immunoassay method approved by the FDA and CE-marked for the detection of anti-HCV antibodies in oral mucosal transudate (OMT), blood, serum or plasma, with a sensitivity and specificity equal or even superior to gold standard EIA/CIA assays [9–12]. In our hands, the clinical sensitivity and of the OraQuick HCV rapid antibody test in OMT was previously established up to 90,1% and 99.1% for past (non-viremic) or current (viremic) HCV infection, respectively; with a clinical specificity of 100% in both cases [13]. The test was performed in OMT at the Health Department’s reference Hospital according to the manufacturer's instructions.

### Follow-up of anti-HCV positive patients

Patients who tested anti-HCV positive with the OraQuick HCV test underwent a serological confirmation test for HCV antibodies in venous blood by a gold-standard immunoassay (COBAS Elecsys® Anti-HCV-II electrochemiluminescence immunoassay Roche diagnostics, Mannheim, Germany). Those with a confirmed positive test were offered appropriate care that included determination of viral genotype (Versant® HCV Genotype LiPA 2.0, Siemens Healthcare Diagnostics, Berkeley, USA), HCV-RNA levels (real-time PCR-based assay Cobas® TaqMan HCV v2.0, Roche Diagnostics, Sant Cugat, Spain) and liver fibrosis stage (elastography and/or liver biopsy), as well as indication for antiviral therapy.

### Statistical analyses

First, the characteristics of the participants were described using means or proportions according to the type of variable, and including their 95% confidence intervals (CI) when appropriate. Then the rates of individuals with anti-HCV antibodies and with active infection, standardized by age for men and women, were estimated separately, along with the rates standardized by age and sex for the whole population (allowing an adjustment in HCV prevalence in line with the different age-sex distribution between the sample and the reference population), and then according to specific characteristics and risk factors of the population with their corresponding CI. Finally, the characteristics of the cases with active infection were compared with those of non-viremic using Fisher’s exact test. All analyses were performed using STATA® software version 13 (StataCorp, College Station, Texas). A minimal anonymized data set is available in the Dryad Digital Repository <https://datadryad.org/> with the doi:10.5061/dryad.9ghx3ffdd.

### Ethics

This study was conducted in accordance with the Declaration of Helsinki and the study protocol was approved by the Research Ethics Committee of the *Hospital Universitario y Politécnico La Fe* (2015, April 7; reference number: 2014/0430). Written informed consent was obtained from all participants prior to their enrolment in the study.

## Results

### Study population

First, we prepared a population sample of 6,000 target adults over 18 years of age, randomly selected by age and sex strata, with the intention of sending them successive waves of invitation letters according to the workloads assumable by the research team. During the first mailshot wave in September, 2015, 300 letters were sent, but only 18 (6%) of the individuals mailed responded agreeing to participate in the study. We speculated that the low response could be due to the recent summer holyday season, but following rates were also was below 10%. Therefore, in the successive mail waves other strategies were tried to improve the response rate, including the incorporation of a pre-determined appointment and direct telephone contact by a doctor.

The incorporation of a pre-determined appointment in the invitation letter (110 letters were sent with pre-determined appointment) procured a response rate of 12.7%, with no substantial increase over the previous strategy and a greater complexity in the management of appointments.

Using a direct telephone contact approach (we attempted to contact 467 people after sending them the corresponding invitation letter), we get to contact to and interview with 178 persons (38.1% of telephoned individuals, details in S1 Table). Only 100 of the interviewed (56.2%) confirmed to have previously received the letter, 53 (29.8%) claimed not to have received the letter, and 25 (14.0%) were not sure. From the telephone interviews, 68 (38.2%) agreed to participate in the study while 110 (61.8%) declined. The most common reasons for declining participation were change of address, schedule incompatibility, elderly person/disability, and refusing to participate in research. Nevertheless, only 53 (11,3% of the 467 telephone attempts) finally approached the Hospital and could be included in the study (23–30.26%- male and 30–29.41%- female).

Due to the discreet success of the recruitment protocol modifications, and because of the increase in the time burden dedicated to phone contacts, we decided to continue with the initial mailing format, but increasing the number of individuals we attempted to contact. and prepared second sample population of 30,000 target adults randomly selected by age and sex strata as the previous sample.

**Fig 1:**
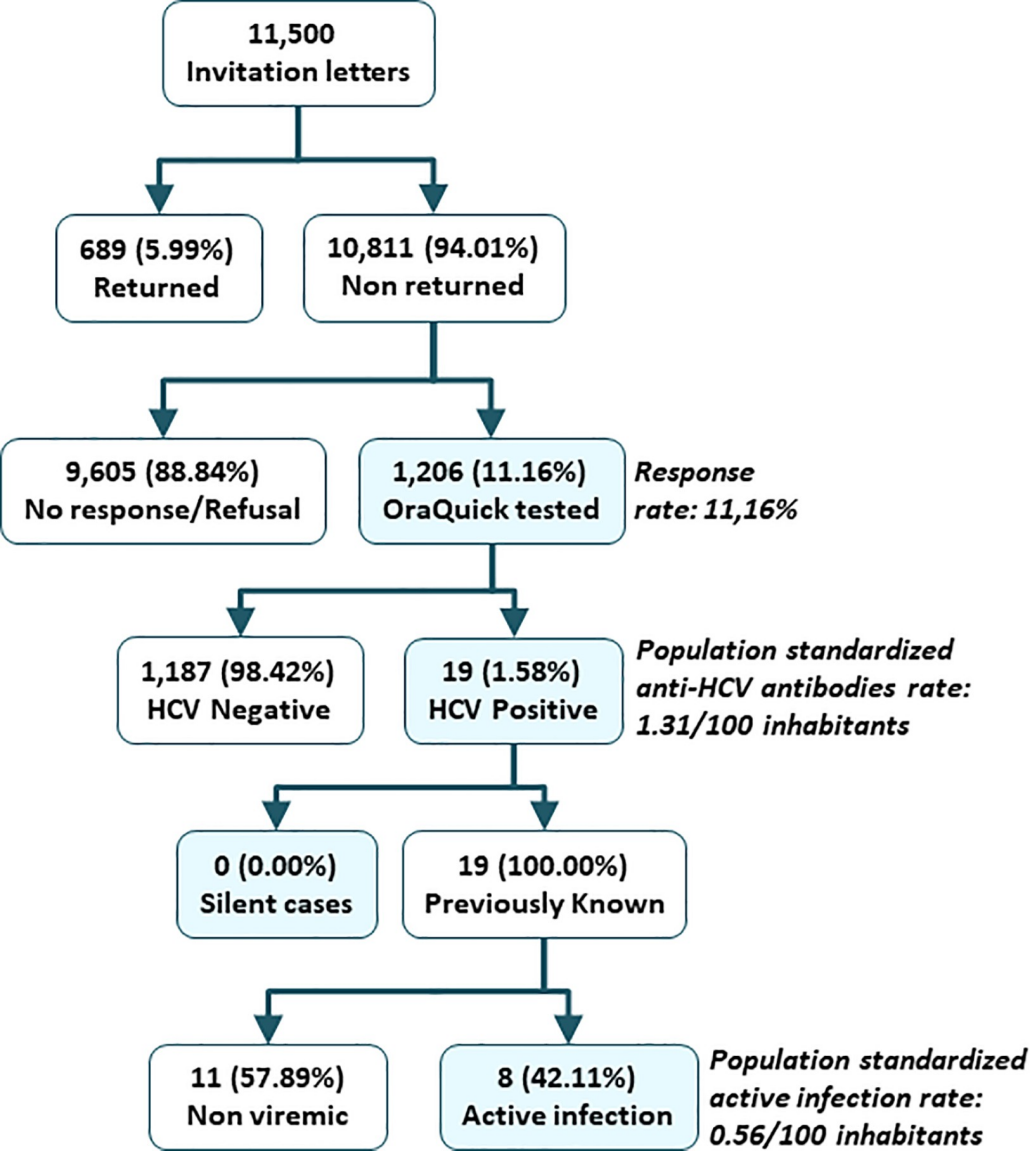
Study diagram. NOTE: HCV: Hepatitis C Virus; Ab: antibodies.

In all, from September 2015 to November 2017 a total of 11,500 invitation letters were sent to the target population. A total of 689 (5.99%) letters were returned to sender (change of address, incomplete, erroneous address or death). From the remaining 10,811 invitations, 1,206 individuals agreed to participate in the study with a final response rate of 11.16% (Fig 1).

The main characteristics of the study participants are summarized in Table 2. The mean age was 55.44 years old (95%CI: 54.61–56.27; male: 57.05 [95%CI: 55.83–58.27]; female: 54.17 [95%CI: 53.05–55.30]), 43.95% were male, 51.99% were born between 1955 and 1964, 86.15% were born in Spain, and 24.42% had at least one risk factor for HCV infection. The age-sex structure of the participants was quite different from the age-sex structure of the hospital district, with the youngest and oldest groups being underrepresented, while the intermediate age groups were overrepresented, especially those between 55 and 75 years of age (S2 Table). Women were also slightly overrepresented with respect to men (S2 Table).

**Table 2:** Characteristics of the study participants.

		*Male*		*Female*		*All*	
		*n*	*%*	*n*	*%*	*n*	*%*
Age group	18-44y	121	22.83	192	28.40	313	25.95
	45-64y	211	39.81	282	41.72	493	40.88
	65-79y	184	34.72	193	28.55	377	31.26
	80+	14	2.64	9	1.33	23	1.91
Birth	<1945	82	15.47	65	9.62	147	12.19
cohort	1945–1965	272	51.32	355	52.51	627	51.99
	>1965	176	33.21	256	37.87	432	35.82
Country	Spain	472	89.06	567	83.88	1,039	86.15
Of birth	Other	58	10.94	109	16.12	167	13.85
Employment	Active	230	43.40	309	45.71	539	44.69
	Unemployed	58	10.94	118	17.46	176	14.59
	Pensioner	237	44.72	231	34.17	468	38.81
	Student	0	0.00	7	1.04	7	0.58
Risk	PWID	3	0.57	3	0.44	6	0.50
factors	HIV Infection	3	0.57	1	0.15	4	0.33
	Haemophilia	0	0.00	0	0.00	0	0.00
	Dialysis	1	0.19	1	0.15	2	0.17
	Transfusion b/1992	28	5.28	45	6.66	73	6.05
	Organ rec. b/ 1992	2	0.38	1	0.15	3	0.25
	Liver enzymes incr.	107	20.19	128	18.93	235	19.49
*n* risk	None	403	76.04	507	75.00	910	75.46
Factors *(a)*	1	112	21.13	155	22.93	267	22.14
	2+	15	2.83	12	1.78	27	2.24
Partner	HCV-	511	96.41	653	96.60	1,164	96.52
	HCV+	17	3.21	20	2.97	37	3.07
Multiple	No	426	80.38	577	85.36	1,003	83.17
Partners	Yes	87	16.42	76	11.24	163	13.52
All *(b)*		530	43.95	676	56.05	1,206	100.00

NOTE: (a) Unweighted sum of the 6 previous risk factors, without excluding cases that did not respond to any factor; (b) Row percentage. HIV: Human Immunodeficiency Virus; HCV: Hepatitis C Virus; PWID: person who injected drugs. Variables with missing data: Employment (n = 16), PWID n = (2), HIV infection (n = 1), haemophilia (n = 3), dialysis n = (9), transfusion before 1992 (n = 19), organ receptor before 1992 (n = 3), number of risk factors (n = 2), partner with HCV+ (n = 5), liver enzymes increase (n = 22), diverse partners (n = 40).

### Prevalence of HCV antibodies as determined with the OraQuick HCV test

Nineteen out of 1,206 sampled individuals (1.58%) had HCV antibodies according to the OraQuick HCV test in OMT, which translates into an age-sex standardized prevalence rate of 1.31 (95%CI: 0.82; 2.07) positive cases by 100 district inhabitants (Table 3). Standardized rates for men (1.18; 95%CI: 0.61; 2.27) and women (1.42; 95%CI: 0.75–2.69) were not significantly different. Standardized rates were significantly higher in older cohorts (2.33 for individuals born between 1945–1965 vs. 0.29 for individuals born after 1965), individuals with risk factors (3.72 and 8.88 for individuals with one or two and more risk factors vs. 0.40 for those with none), and partners of individuals with anti-HCV antibodies (9.41 vs. 1.06). Non-significant differences were present in the oldest age groups (2.50 and 2.95 vs. 0.51 for 65–79, 80 and over and under 45 years old groups, respectively), individuals born in Spain (1.41 vs. 0.82 for born abroad), those active and unemployed (2.25 and 2.09, vs. 1.21 for pensioners) and those citing multiple partners (2.72 vs. 1.42). Differences between men and women were non-significant in all stratified analyses.

**Table 3:** Anti-HCV antibodies and active infection prevalence. Standardized rates by 100 inhabitants.

		*Male*			*Female*			*All*		
		*HCVpos*	*Rate*	*95%CI*	*HCVpos*	*Rate*	*95%CI*	*HCVpos*	*Rate*	*95%CI*
***Anti-HCV prevalence***										
Age	18-44y	0/121	0.00	---	2/192	1.04	0.26; 4.09	2/313	0.51	0.12; 2.02
group	45-64y	5/211	2.37	0.99; 5.59	2/282	0.71	0.18; 2.80	7/493	1.50	0.72; 3.12
	65-79y	3/184	1.63	0.52; 4.96	6/193	3.11	1.40; 6.77	9/377	2.50	1.29; 4.76
	80+	1/14	7.14	0.92;38.95	0/9	0.00	---	1/23	2.95	0.41;18.23
Birth	<1945	1/82	1.22	0.17; 8.27	2/65	3.08	0.76;11.62	3/147	2.31	0.72; 7.15
year	1945–1965	8/272	2.98	1.48; 5.83	6/355	1.80	0.81; 3.96	14/627	2.33	1.38; 3.90
	>1965	0/176	0.00	---	2/256	0.58	0.15; 2.31	2/432	0.29	0.07; 1.17
Country	Spain	8/472	1.10	0.54; 2.22	10/567	1.69	0.86; 3.28	18/1.039	1.41	0.86; 3.31
of birth	Other	1/58	1.72	0.24;11.07	0/109	0.00	---	1/167	0.82	0.12; 5.38
Employm.	Active	1/230	0.24	0.03; 1.69	5/287	4.08	1.54;10.33	6/546	2.25	0.90; 5.53
	Unempl.	2/58	2.63	0.67; 9.80	2/118	1.70	0.43; 6.49	4/176	2.09	0.80; 5.38
	Pensioner	6/237	2.05	0.68; 6.03	3/231	0.44	0.14; 1.34	9/468	1.21	0.48; 2.98
Number	None	2/403	0.26	0.06; 1.02	3/507	0.54	0.17; 1.65	5/910	0.40	0.17; 0.98
of risk	1	5/112	2.68	1.12; 6.26	4/155	4.67	1.51;13.55	9/267	3.72	1.68; 8.03
factors	2+	2/15	6.53	1.73;21.66	3/12	11.01	4.33;25.28	5/27	8.88	4.24;17.67
Partner	HCV(-)	6/511	0.91	0.41; 2.02	8/653	1.20	0.59; 2.45	14/1.164	1.06	0.62; 1.81
	HCV(+)	2/17	7.56	2.01;24.68	2/20	10.28	3.24;28.16	4/37	9.41	3.82;21.33
Multiple	No	8/426	1.30	0.64; 2.60	9/577	1.52	0.76; 3.04	17/1.003	1.42	0.82; 2.32
Partners	Yes	1/87	1.00	0.14; 6.57	1/76	4.28	6.75;22.77	2/163	2.72	0.58;11.77
All		9/530	1.18	0.61; 2.27	10/676	1.42	0.75; 2.69	19/1.206	1.31	0.82; 2.07
***Active infection prevalence (HCV-RNA)***										
All		2/530	0.26	0.06; 1.05	6/676	0.84	0.37; 1.91	8/1.026	0.56	0.28; 1.14

**NOTE:** HCVpos = anti-HCV positive. Age standardization for male and female. and age and sex standardization for all population. Variables with missing data: employment (n = 16). number of risk factors (n = 2). partner with HCV+ (n = 5). multiple partners (n = 40).

All anti-HCV positive individuals were already aware of their infection, but three of them had previously abandoned medical (hepatology) monitoring (after screening they were reincorporated into the appropriate care schemes). The OraQuick HCV rapid test failed to detect two patients with a past positive result for anti-HCV antibodies. These two patients had been infected in the past (>10 years before). According chart evaluation one had cleared the virus spontaneously while the other had cleared the virus after successful antiviral treatment with interferon plus ribavirin.

Individuals with a positive OraQuick HCV test in OMT were prospectively assessed by a hepatologist. Chart review together with new HCV-RNA testing (limit of detection 15 IU/mL) indicated that, of the 19 OraQuick HCV positive cases, 8 (42.1%) were patients with active infection and 11 (57.9%) were non-viremic. These results translate into an age-sex standardized prevalence rate for active infection of 0.56 by 100 district inhabitants (95%CI: 0.28; 1.14). Women (0.84, 95%CI: 0.37; 1.91) showed higher rates of active infection than men (0.26, 95%CI: 0.06; 1.05), but differences were not significant.

### Characteristics of individuals with a positive OraQuick HCV test result

The HCV genotype distribution together with stage of fibrosis and baseline features of OraQuick positive patients (both HCV-RNA positive and negative) are shown in Table 4. No substantial differences were found regarding viral genotype distribution, baseline features or stage of fibrosis.

**Table 4:** Characteristics of the Anti-HCV positive patients.

		*Non Viremic* *(n = 11)*		*Active infection* *(n = 8)*		*Total Anti-HCV* *(n = 19)*		
		n	%	n	%	n	%	*p*
Gender	Male	7	63.6	2	25.0	9	47.4	*0*.*170*
	Female	4	36.4	6	75.0	10	52.6	
Genotype	1a	2	18.2	1	12.5	3	15.8	*1*.*000*
	1b	5	45.4	4	50.0	9	47.3	
	2	0	0.0	1	12.5	1	5.3	
	3	1	9.1	0	0.0	1	5.3	
	5a	1	9.1	1	12.5	2	10.5	
Viral	Spontaneous	1	9.1	0	0.0	1	5.3	*0*.*512*
Clearance	IFN	1	9.1	0	0.0	1	5.3	
	IFN+RBV	2	18.2	0	0.0	2	10.5	
	DAA	7	63.6	7 (*a*)	87.5	14	73.7	
Fibrosis	F0-F2	5	45.5	4	50.0	9	47.4	*1*.*000*
	F3-F4	5	45.5	3	37.5	8	42.1	
Risk	Yes	7	63.6	7	87.5	14	73.7	*0*.*338*
Factors	No	4	36.4	1	12.5	5	26.3	

NOTE: (a) Treatment started after OraQuick HCV testing: five patients already in the system; three patients relinked to the system after participating in the study (2 DAA treatment. and 1 awaiting fibrosis evaluation). (b) Variables with missing data: Genotype (n = 3). viral clearance (n = 1). fibrosis (n = 2). (c) IFN = interferon. RBV = ribavirin; F = fibrosis. DAA = direct acting antivirals

Of the non-viremic individuals, one had spontaneously resolved the infection 15 years before OraQuick HCV testing, one had cleared the virus after interferon treatment 23 years before OraQuick HCV testing, two had cleared the virus following interferon and ribavirin treatment 6 and 16 years before OraQuick HCV testing, while 7 had achieved a sustained virological response (SVR) after receiving a course of direct-acting antiviral (DAA) therapy at a median of 23 months before OraQuick testing. All the 8 viremic patients were also aware of their infection; five were actively being followed up by a specialist and have been now treated with DAA agents. Regarding the other three patients previously lost for follow up, two have started therapy with DAA agents while the last one is awaiting new evaluation of liver fibrosis.

## Discussion

To our knowledge, this is the first population-based universal screening study in Spain randomly sampling the total population in the catchment area. The most relevant findings of our study show: 1) a low effectiveness of recruitment based on ordinary mail with a participation rate of only 11%; 2) a good performance of the OraQuick HCV rapid test in OMT; 3) a population prevalence of anti-HCV antibodies of 1.31% and of 0.56% for active infection; and 4) all anti-HCV positive patients were aware of their infection, although 16% had left follow-up.

Our recruiting strategy by regular mail showed to be poorly effective, despite trying several changes in the recruitment process. Using a direct telephone contact approach gave us the opportunity to ascertain if the letters reached the target individuals, and to explore the potential reasons of declining participation. Only 56.2% of the interviewed confirmed to have received the letter, suggesting an actual loss of 43.8% of the target. From the 38% who agreed to participate in the study by the phone, 30% finally approached the Hospital and could be included in the study. Some of the justifications given for declining to cooperate by those interviewed were change of address, schedule incompatibility, elderly person/disability, advanced age and/or disability and refusing to participate in research mainly due to a healthy status with lack of interest in the screening proposal. Despite the increased response rates, the time burden imposed by telephone interviewing and citation no-shows precluded extension of this approach to all potential participants. In line with our results, another study in Barcelona, Spain, based on invitation for a blood HCV test by ordinary mail, showed an even lower response rate (4.11%: 238 participants out of 5,793 letters sent) [18]. Successful recruitment in this type of population studies probably requires different approaches and better communication strategies, including the use of the media and social networks, especially in younger populations and in urban areas [19].

The performance of the OraQuick HCV rapid test was excellent for detecting active HCV infection. Because the aim of screening is to detect new HCV infected individuals with active viral replication, testing OMT with the OraQuick HCV rapid test seems sufficiently sensitive. There were 21 individuals with a previous anti-HCV-positive laboratory record (9.5%), but the OraQuick HCV rapid test failed to detect two of them. These two “false-negatives” in fact corresponded to spontaneous or treatment-induced HCV clearance, achieved more than 24 months before OraQuick testing. However, it has been reported that the test sometimes fail in OMT samples from individuals with an altered immune response or under immunosuppression.[13,20–22] In these reports, the test sensitivity raised to 94.7% in OMT when reading was taken after 40 minutes, and up to 99.4% when using fingerstick blood.[13,20,21]

Several studies, mainly from the USA, suggest that chronic HCV hepatitis is an under-diagnosed disease and that the proportion of undiagnosed individuals is higher than that of those diagnosed with HCV infection.[23] In contrast, in our study only 19 of the 1,206 individuals tested showed a positive OraQuick HCV test, and all were aware of their infection. The population prevalence of anti-HCV antibodies as detected with the OraQuick HCV test was 1.31%, and the prevalence of active HCV-RNA positive infection was only 0.56%, both figures lower than expected according to the Spanish HCV Strategic Plan [estimated figures of 1.7% (CI: 0.4; 2.6) for anti-HCV prevalence and 1.2% (CI: 0.3; 1.8) for active infection prevalence],[6] but in line with recent studies from other regions in Spain on selected populations reporting low anti-HCV estimates in patients scheduled for elective surgery, [24] colorectal cancer screening,[25] or recruited for a seroepidemiological, [26] or hepatitis surveys. [27] Recent studies from other Western European countries also show prevalence rates below 1%.[28,29]

The absence of undiagnosed cases in our study (i.e. individuals unaware of their HCV infection) is at odds with studies from the USA,[23] but consistent with some studies in Spain.[24] Although it is possible that individuals with known risk factors for HCV were less likely to participate, our results are probably related to the free, universal, public healthcare Spanish system and the National Hepatitis C Plan implemented in 2011, where free access to new DAA was offered, first to patients with advanced fibrosis and more recently to all HCV infected individuals.

Several limitations of our study may hamper the generalization of our HCV prevalence rates beyond our region. First, our power calculations were based in a 2% HCV prevalence and 50% response rate, but we finally obtained a response rate of only 11%. Although the estimated anti-HCV prevalence was lower than expected (1.31%), our study is potentially underpowered. In addition, we cannot exclude the existence of a population bias due to the dominance of a “relatively low risk” population on the census from our Health Care Department, with no prisons or districts with drug use problems belonging to the assigned healthcare area. Thus, difficult to reach populations such as homeless and PWID are probably underrepresented in our study (i.e. there were only 0.5% of PWID in the overall screened population). Finally, almost ninety percent of the individuals contacted did not respond to the survey and we have no information regarding their baseline characteristics.

Despite these limitations, our study suggests a low prevalence of HCV in the Department District and the relatively advanced age of the anti-HCV positive population, in line with previous studies in Spain.[1,24,30] The age-cohort with most OraQuick HCV positive cases was that of 60–69 years old (42.1%); and 73.7% of the cases occurred in individuals born between 1945 and 1965, with 89.5% of anti-HCV positive patients being born before 1965. In addition, although anti-HCV positive individuals had a higher prevalence of risk factors compared to those who tested negative, no new diagnoses were made through population screening. This finding is relevant because the value of the screening programs lies in the detection of previously unknown active cases, and our study indicates a very low performance of population screening for this purpose. Collectively, our results–and other data from Spain and other European countries–suggests a different epidemiological situation than in other countries that recommend to screen all individuals born between 1945 and 1965 and/or some groups of those considered to be at high risk of HCV infection.[24,30–37] Perhaps, and in light of the few previously undiagnosed cases detected by this type of screening, the performance of other strategies should be re-evaluated, including “opportunistic screening” or “case finding” schemes, whereby patients from vulnerable populations and patients attending care who have risk factors are actively tested, retrieval policies to re-evaluate VHC patients lost to follow-up and targeting specific subgroups (i.e. methadone clinic attendees).

## Supporting information

S1 Table Characteristics of the individuals interviewed by direct telephone contact. (DOCX)

S2 Table Distribution by age groups and sex of the Valencia La Fe Healthcare Department population and the study sample. (DOCX)

## References

[1] Gower E, Estes C, Blach S, Razavi-Shearer K, Razavi H. Global epidemiology and genotype distribution of the hepatitis C virus infection. J Hepatol. 2014;61(Suppl1):S45–S57. 25086286 10.1016/j.jhep.2014.07.027

[2] Shepard CW, Finelli L, Alter MJ. Global epidemiology of hepatitis C virus infection. Lancet Inf Dis. 2005;5:558–567. 10.1016/S1473-3099(05)70216-4 16122679

[3] Blach S, Zeuzem S, Manns M, Altraif I, Duberg AS, Muljono DH, et al Global prevalence and genotype distribution of hepatitis C virus infection in 2015: a modelling study. Lancet Gastroenterol Hepatol. 2017;2(3):161–176. 10.1016/S2468-1253(16)30181-9 28404132

[4] World Health Organization. Global Hepatitis Report, 2017; 2017. ISBN 978-92-4-156545-5. Available at: http://www.who.int/hepatitis/publications/global-hepatitis-report2017/en/

[5] Duffell EF, Van De Laar MJW, Amato-Gauci AJ. Enhanced surveillance of hepatitis C in the EU, 2006–2012. J Viral Hepat. 2015;22(7):590–595. 10.1111/jvh.12367 25420699

[6] Ministerio de Sanidad SS e Igualdad, Sanidad DE, Sociales S, Igualdad E. Plan estratégico para el abordaje de la hepatitis c en el sistema nacional de salud. Ministerio de Sanidad, Servicios Sociales e Igualdad. https://www.msssi.gob.es/ciudadanos/enfLesiones/enfTransmisibles/docs/plan_estrategico_hepatitis_C.pdf. Published 2015.

[7] Faustini A, Colais P, Fabrizi E, Bargagli AM, Davoli M, Di Lallo D, et al Hepatic and extra-hepatic sequelae, and prevalence of viral hepatitis C infection estimated from routine data in at-risk groups. BMC Infect Dis. 2010;10(1):97. 20403169 10.1186/1471-2334-10-97 PMC2867994

[8] Zuure FR, Urbanus AT, Langendam MW, Helsper CW, van den Berg CH, Davidovich U, et al Outcomes of hepatitis C screening programs targeted at risk groups hidden in the general population: a systematic review. BMC Public Health. 2014;14(1):66. 24450797 10.1186/1471-2458-14-66 PMC4016146

[9] Lee SR, Yearwood GD, Guillon GB, Kurtz LA, Fischl M, Friel T, et al Evaluation of a rapid, point-of-care test device for the diagnosis of hepatitis C infection. J Clin Virol. 2010;48(1):15–17. 10.1016/j.jcv.2010.02.018 20362493

[10] Lee SR, Kardos KW, Schiff E, Berne CA, Mounzer K, Banks AT, et al Evaluation of a new, rapid test for detecting HCV infection, suitable for use with blood or oral fluid. J Virol Methods. 2011;172(1–2):27–31. 10.1016/j.jviromet.2010.12.009 21182871

[11] Cha YJ, Park Q, Kang ES, Yoo BC, Park KU, Kim JW, et al Performance evaluation of the oraquick hepatitis C virus rapid antibody test. Ann Lab Med. 2013;33(3):184–189. 10.3343/alm.2013.33.3.184 23667844 PMC3646192

[12] Drobnik A, Judd C, Banach D, Egger J, Konty K, Rude E. Public health implications of rapid hepatitis C screening with an oral swab for community-based organizations serving high-risk populations. Am J Public Health. 2011;101(11):2151–2155. 10.2105/AJPH.2011.300251 21940910 PMC3222414

[13] Pallarés C, Carvalho-Gomes Â, Hontangas V, Conde I, Di Maira T, Aguilera V, et al Performance of the OraQuick HCV rapid test in oral fluid and fingerstick blood in patients with liver disease and concordance with Hepatitis C virus antibody. J Clin Virology 2018;102:77–83. 29525634 10.1016/j.jcv.2018.02.016

[14] Jewett A, Al-Tayyib AA, Ginnett L, Smith BD. Successful integration of hepatitis C virus point-of-care tests into the Denver Metro Health Clinic. AIDS Res Treat. 2013;2013. 10.1155/2013/528904 PMC3881337 24455220

[15] Ibrahim S, Al Attas SA, Mansour GA, Ouda S, Fallatah H. Accuracy of rapid oral HCV diagnostic test among a Saudi sample. Clin Oral Investig. 2015;19(2):475–480. 10.1007/s00784-014-1261-y 24846644

[16] Hayes B, Briceno A, Asher A, Yu M, Evans JL, Hahn JA, et al Preference, acceptability and implications of the rapid hepatitis C screening test among high-risk young people who inject drugs. BMC Public Health. 2014;14(1):645. 24965699 10.1186/1471-2458-14-645 PMC4091768

[17] Judd A, Parry J, Hickman M, McDonald T, Jordan L, Lewis K, et al Evaluation of a modified commercial assay in detecting antibody to hepatitis C virus in oral fluids and dried blood spots. J Med Virol. 2003;71(1):49–55. 10.1002/jmv.10463 12858408

[18] Caballería L, Pera G, Bernad J, Canut S, Navarro E, Bruguera M. Strategies for the Detection of Hepatitis C Viral Infection in the General Population. Rev ClinEsp (Barc). 2014;214(5):242–6. 10.1016/j.rce.2014.01.024 24598246

[19] Parda N, Stępień M, Zakrzewska K, Madaliński K, Kołakowska A, Godzik P, et al What affects response rates in primary healthcare-based programmes? An analysis of individual and unit-related factors associated with increased odds of non-response based on HCV screening in the general population in Poland. BMJ Open 2016;6:e013359 10.1136/bmjopen-2016-013359 27927665 PMC5168657

[20] Kosack CS, Nick S. Evaluation of two rapid screening assays for detecting hepatitis C antibodies in resource-constrained settings. Trop Med Int Heal. 2016;21(5):603–609. 10.1111/tmi.12688 26945920

[21] Campbell JI, Kanters S, Bennett JE, Thorlund K, Tsai AC, Mills EJ, et al Comparative Effectiveness of Induction Therapy for Human Immunodeficiency Virus-Associated Cryptococcal Meningitis: A Network Meta-Analysis. Ofid. 2015;2(Suppl 1):1–8. 10.1093/ofid/ofv010 PMC4438891 26034761

[22] Smith BD, Teshale E, Jewett A, Weinbaum CM, Neaigus A, Hagan H, et al Performance of premarket rapid hepatitis C virus antibody assays in 4 national human immunodeficiency virus behavioral surveillance system sites. Clin Infect Dis. 2011;53(8):780–786. 10.1093/cid/cir499 21921221

[23] Chak E, Talal AH, Sherman KE, Schiff ER, Saab S. Hepatitis C virus infection in USA: An estimate of true prevalence. Liver Int. 2011;31(8):1090–1101. 10.1111/j.1478-3231.2011.02494.x 21745274

[24] Aguinaga A, Díaz-González J, Pérez-García A, Barrado L, Martínez-Baz I, Casado I, et al The prevalence of diagnosed and undiagnosed hepatitis C virus infection in Navarra, Spain, 2014–2016. Enferm Infecc Microbiol Clin. 2018; 36(6):325–331. 10.1016/j.eimc.2016.12.008 28110858

[25] García-Alonso FJ, Bonillo-Cambrodón D, Bermejo A, García-Martínez J, Hernández-Tejero M, Valer López Fando P, et al Acceptance, yield and feasibility of attaching HCV birth cohort screening to colorectal cancer screening in Spain. Dig Liver Dis. 2016;48(10):1237–1242. 10.1016/j.dld.2016.06.034 27481585

[26] Departamento de Sanidad y Consumo. I Encuesta de Seroprevalencia de la Comunidad Autónoma del País Vasco. Vitoria-Gasteiz: Servicio Central de Publicaciones del Gobierno Vasco. 2011. [Accessed in February 7, 2018]. Available in http://www.euskadi.eus/contenidos/informacion/publicaciones_departamento/es_def/adjuntos/salud_publica/seroprevalencia.pdf.

[27] Cuadrado A, Perelló C, Llerena S, Escudero MD, Gómez M, Estébanez Á, et al Diseño y coste-efectividad de una política de eliminación del VHC basada en un studio epidemiológico actualizado (Cohorte ETHON). Gastroenterol Hepatol. 2018; 41 (suppl):4–5.

[28] Parisi MR, Tecco S, Gastaldi G, Polizzi E, D'Amicantonio T, Negri S, et al Point-of-care testing for hepatitis C virus infection at alternative and high-risk sites: an Italian pilot study in a dental clinic. New Microbiol. 2017; 40(4):242–245. 28825443

[29] Carvalhana SC, Leitão J, Alves AC, Bourbon M, Cortez-Pinto H. Hepatitis B and C prevalence in Portugal: Disparity between the general population and high-risk groups. Eur J Gastroenterol Hepatol. 2016;28(6):640–644. 10.1097/MEG.0000000000000608 26866523

[30] García Comas L, Ordobás Gavín M, Sanz Moreno JC, Ramos Blázquez B, Gutiérrez Rodríguez A, Astray Mochales J, et al Prevalence of hepatitis C antibodies in the population aged 16–80 years in the Community of Madrid 2008–2009. J Med Virol. 2015;87(10):1697–1701. 10.1002/jmv.24219 25989026

[31] Smith BD, Morgan RL, Beckett G, Falck-Ytter Y, Holtzman D, Teo CG, et al Recommendations for the identification of chronic hepatitis C virus infection among persons born during 1945–1965. Morb Mortal Wkly Rep. 2012;61(RR-4):1–32. 22895429

[32] Myers RP, Krajden M, Bilodeau M, Kaita K, Marotta P, Peltekian K, et al Burden of Disease and Cost of Chronic Hepatitis C Virus Infection in Canada. Can J Gastroenterol Hepatol. 2014;28(5):243–250. 10.1155/2014/317623 24839620 PMC4049256

[33] Averhoff FM, Glass N, Holtzman D. Global burden of hepatitis C: considerations for healthcare providers in the United States. Clin Infect Dis. 2012;55 Suppl 1(Suppl 1):10–15. 10.1093/cid/cis361 22715208

[34] Rein D, Smith B, Wittenborn J. The Cost-Effectiveness of Birth Cohort Screening for Hepatitis C Antibody in US Primary Care Settings. Gastroenterology. 2013;144(2):457–459. 10.1053/j.gastro.2012.12.013 23260498

[35] Shah HA, Heathcote J, Feld JJ. A Canadian screening program for hepatitis C: Is now the time? CMAJ. 2013;185(15):1325–1328. 10.1503/cmaj.121872 24082023 PMC3796597

[36] Allison WE, Chiang W, Rubin A, O'Donnell L, Saldivar MA, Maurantonio M, et al Hepatitis C virus infection in the 1945–1965 birth cohort (baby boomers) in a large urban ED. Am J Emerg Med. 2016;34(4):697–701. 10.1016/j.ajem.2015.12.072 26809931

[37] Allison WE, Chiang W, Rubin A, Oshva L, Carmody E. Knowledge about Hepatitis C Virus Infection and Acceptability of Testing in the 1945–1965 Birth Cohort (Baby Boomers) Presenting to a Large Urban Emergency Department: A Pilot Study. J Emerg Med. 2016;50(6):825–831. 10.1016/j.jemermed.2016.02.001 26954104

