# Supplementary material for: A population-based screening for hepatitis C antibodies and active infection using a point-of-care test in a low prevalence area
Source: PLoS One. 2020 Feb 11;15(2):e0228351. doi: 10.1371/journal.pone.0228351 (PMC7012430; doi:10.1371/journal.pone.0228351)
Supplement: S1 Table — (DOCX) [file pone.0228351.s001.docx]

**A population-based screening for hepatitis C antibodies and active infection using a point-of-care test in a low prevalence area.**

**Ângela Carvalho-Gomes et al.**

**Supplementary table 1. Characteristics of the individuals interviewed by direct telephone contact.**

| **.** |  | ***Male*** | | ***Female*** | | ***All*** | |  |
| --- | --- | --- | --- | --- | --- | --- | --- | --- |
|  |  | ***n*** | ***%*** | ***n*** | ***%*** | ***n*** | ***%*** |  |
| Age group | 18-44y | 34 | 47.89 | 43 | 42.57 | 77 | 43.3 |  |
|  | 45-64y | 16 | 22.54 | 24 | 23.76 | 40 | 22.5 |  |
|  | 65-79y | 14 | 19.72 | 21 | 20.79 | 35 | 19.7 |  |
|  | 80+ | 7 | 9.86 | 13 | 12.87 | 20 | 11.2 |  |
| Birth | <1945 | 16 | 22.54 | 23 | 22.77 | 39 | 21.9 |  |
| Cohort | 1945-1965 | 12 | 16.9 | 23 | 22.77 | 35 | 19.7 |  |
|  | >1965 | 43 | 60.56 | 55 | 54.46 | 98 | 55.1 |  |
| Country | Spain | 68 | 89.47 | 79 | 77.45 | 147 | 82.5 |  |
| Of birth | Other  NA | 6  2 | 7.89  2.63 | 23  0 | 22.55  0 | 29  2 | 16.2  1.1 |  |
| Received | Yes | 41 | 53.95 | 59 | 57.84 | 100 | 56.2 |  |
| Letter | No | 24 | 31.58 | 29 | 28.43 | 53 | 29.8 |  |
|  | Not sure | 11 | 14.47 | 14 | 13.73 | 25 | 14.0 |  |
| Accept to | Yes | 30 | 39.47 | 38 | 37.25 | 68 | 38.2 |  |
| Participate | No | 46 | 60.53 | 64 | 62.75 | 110 | 61.8 |  |
| Reasons for declining | Change of address | 6 | 13.04 | 8 | 12.50 | 14 | 12.7 |  |
|  | Schedule incompatibility | 13 | 28.26 | 13 | 20.31 | 26 | 23.6 |  |
|  | Feels fine / not considered to be at risk | 2 | 4.35 | 4 | 6.25 | 6 | 5.5 |  |
|  | Elderly person/disability | 12 | 26.09 | 24 | 37.50 | 36 | 32.7 |  |
|  | Don't want to participate in research | 9 | 19.57 | 8 | 12.50 | 17 | 15.5 |  |
|  | Deceased | 3 | 6.52 | 1 | 1.56 | 4 | 3.6 |  |
|  | Distance from the hospital | 2 | 4.35 | 6 | 9.38 | 8 | 7.3 |  |
|  | Have recently been tested | 2 | 4.35 | 2 | 3.13 | 4 | 3.6 |  |
|  | Other | 4 | 8.70 | 9 | 14.06 | 13 | 11.8 |  |
| Performed test | Yes | 23 | 30.26 | 30 | 29.41 | 53 | 29.8 |  |
|  | No | 53 | 69.74 | 72 | 70.59 | 125 | 70.2 |  |
|  | All (row percentages) | 76 | 42.70 | 102 | 57.30 | 178 | 100 |  |
|  | | | | | | | | |
