# Supplementary material for: A population-based screening for hepatitis C antibodies and active infection using a point-of-care test in a low prevalence area
Source: PLoS One. 2020 Feb 11;15(2):e0228351. doi: 10.1371/journal.pone.0228351 (PMC7012430; doi:10.1371/journal.pone.0228351)
Supplement: S2 Table — (DOCX) [file pone.0228351.s002.docx]

**Supplementary Table 2.** Distribution by age groups and sex of the Valencia La Fe Healthcare Department population and the study sample.

|  |  | Population | | Sample | |
| --- | --- | --- | --- | --- | --- |
| Sex | Age group | n | % | n | % |
| Men | 18-24y | 6,907 | 4.51% | 6 | 0,50% |
|  | 25-29y | 5,452 | 3.56% | 12 | 1,00% |
|  | 30-34y | 6,744 | 4.40% | 24 | 1,99% |
|  | 35-39y | 7,882 | 5.14% | 34 | 2,82% |
|  | 40-44y | 7,650 | 4.99% | 45 | 3.73% |
|  | 45-49y | 6,970 | 4.55% | 40 | 3.32% |
|  | 50-54y | 6,356 | 4.15% | 40 | 3.32% |
|  | 55-59y | 5,562 | 3.63% | 69 | 5.72% |
|  | 60-64y | 4,948 | 3.23% | 62 | 5.14% |
|  | 65-69y | 4,655 | 3.04% | 83 | 6.88% |
|  | 70-74y | 3,701 | 2.41% | 66 | 5.47% |
|  | 75-79y | 2,596 | 1.69% | 35 | 2.90% |
|  | 80+ | 3,583 | 2.34% | 14 | 1.16% |
|  | Total | 73,006 | 47.62% | 530 | 43.95% |
| Women | 18-24y | 6,595 | 4.30% | 26 | 2.16% |
|  | 25-29y | 5,447 | 3.55% | 23 | 1.91% |
|  | 30-34y | 6,587 | 4.30% | 32 | 2.65% |
|  | 35-39y | 7,537 | 4.92% | 47 | 3.90% |
|  | 40-44y | 7,312 | 4.77% | 64 | 5.31% |
|  | 45-49y | 6,997 | 4.56% | 55 | 4.56% |
|  | 50-54y | 6,903 | 4.50% | 60 | 4.98% |
|  | 55-59y | 6,409 | 4.18% | 75 | 6.22% |
|  | 60-64y | 5,885 | 3.84% | 92 | 7.63% |
|  | 65-69y | 5,507 | 3.59% | 106 | 8.79% |
|  | 70-74y | 4,673 | 3.05% | 58 | 4.81% |
|  | 75-79y | 3,736 | 2.44% | 29 | 2.40% |
|  | 80+ | 6,724 | 4.39% | 9 | 0.75% |
|  | Total | 80,312 | 52.39% | 676 | 56.07% |
| All | 18-24y | 13,502 | 8.81% | 32 | 2.65% |
|  | 25-29y | 10,899 | 7.11% | 35 | 2.90% |
|  | 30-34y | 13,331 | 8.70% | 56 | 4.64% |
|  | 35-39y | 15,419 | 10.06% | 81 | 6.72% |
|  | 40-44y | 14,962 | 9.76% | 109 | 9.04% |
|  | 45-49y | 13,967 | 9.11% | 95 | 7.88% |
|  | 50-54y | 13,259 | 8.65% | 100 | 8.29% |
|  | 55-59y | 11,971 | 7.81% | 144 | 11.94% |
|  | 60-64y | 10,833 | 7.07% | 154 | 12.77% |
|  | 65-69y | 10,162 | 6.63% | 189 | 15.67% |
|  | 70-74y | 8,374 | 5.46% | 124 | 10.28% |
|  | 75-79y | 6,332 | 4.13% | 64 | 5.31% |
|  | 80+ | 10,307 | 6.72% | 23 | 1.91% |
|  | Total | 153,318 | 100.00% | 1,206 | 100.00% |
